# A Fiber-Optic Sensor Using an Aqueous Solution of Sodium Chloride to Measure Temperature and Water Level Simultaneously

**DOI:** 10.3390/s141018823

**Published:** 2014-10-10

**Authors:** Wook Jae Yoo, Hyeok In Sim, Sang Hun Shin, Kyoung Won Jang, Seunghyun Cho, Joo Hyun Moon, Bongsoo Lee

**Affiliations:** 1 School of Biomedical Engineering, College of Biomedical & Health Science, BK21 Plus Research Institute of Biomedical Engineering, Konkuk University, 268 Chungwon-daero, Chungju-si, Chungcheongbuk-do 380-701, Korea; E-Mails: wonzip@kku.ac.kr (W.J.Y.); saucony9116@gmail.com (H.I.S.); shshin9431@gmail.com (S.H.S.); kko988@kku.ac.kr (K.W.J.); 2 Department of Organic Materials & Fiber Engineering, College of Engineering, Soongsil University, 369 Sangdo-ro, Dongjak-gu, Seoul-si 156-743, Korea; E-Mail: scho@ssu.ac.kr; 3 Department of Nuclear & Energy System Engineering, College of Natural Science, Dongguk University-Gyeongju, 123 Dongdae-ro, Gyeongju-si, Gyeongsangbuk-do 780-714, Korea; E-Mail: jhmoon86@dongguk.ac.kr

**Keywords:** fiber-optic sensor, NaCl solution, OTDR, temperature, water level

## Abstract

A fiber-optic sensor system using a multiplexed array of sensing probes based on an aqueous solution of sodium chloride (NaCl solution) and an optical time-domain reflectometer (OTDR) for simultaneous measurement of temperature and water level is proposed. By changing the temperature, the refractive index of the NaCl solution is varied and Fresnel reflection arising at the interface between the distal end of optical fiber and the NaCl solution is then also changed. We measured the modified optical power of the light reflected from the sensing probe using a portable OTDR device and also obtained the relationship between the temperature of water and the optical power. In this study, the water level was simply determined by measuring the signal difference of the optical power due to the temperature difference of individual sensing probes placed inside and outside of the water. In conclusion, we demonstrate that the temperature and water level can be obtained simultaneously by measuring optical powers of light reflected from sensing probes based on the NaCl solution. It is anticipated that the proposed fiber-optic sensor system makes it possible to remotely monitor the real-time change of temperature and water level of the spent fuel pool during a loss of power accident.

## 1. Introduction

Spent fuel is highly radioactive as a high-level waste despite that it has been burned in the nuclear reactor. The decay of spent fuel generates heat and penetrating radiation. Therefore, cooling and shielding are required for spent fuel. After nuclear fuel is unloaded from the reactor, it is stored in a spent fuel pool (SFP) to prevent criticality accidents. The depth of the typical SFP is about 12 m and the height of the storage rack is about 4 m. There is about 8 m of water above the top of the storage racks to provide substantial radiation shielding and cooling even when a spent fuel assembly is being moved above the rack [1]. In the SFP, the water level is maintained at more than 7 m above the top of the spent fuel. In addition, the temperature of the water is controlled by an automatic water cooling system to below 45∼50 °C during normal operation [2]. As mentioned above, the spent fuel should be cooled to prevent heat-up to high temperatures from radioactive decay because it still continues to produce decay heat. Therefore, reliable real-time monitoring is essential to measure safety related parameters, such as temperature and water level, inside the SFP [3,4].

Conventionally, thermocouples and resistance temperature detectors (RTDs) are used to measure the temperature of the water and coolant in nuclear facilities, including the SFP, vessel, and piping. In the case of the water level inside the SFP, it is normally measured by using an ultrasonic water level meter. However, there are difficulties in measuring temperature and water level using existing electronic sensors due to their limit of measurable distance, and high electromagnetic interference (EMI) or radiofrequency interference (RFI). In addition, the conventional sensors installed in nuclear facilities cannot operate when it is not possible to closely access the SFP during a loss of power accident because they usually require an alternating current (AC) power supply. To overcome these problems, it is necessary to develop a new concept for remote sensors that can monitor the real-time change of the key environment variables of the inaccessible SFP during a loss of power accident [3,4].

Alternatively, optical fiber-based sensors may be used to measure a variety of physical parameters. For application in nuclear facilities, there have been many studies in recent years on the development of various fiber-optic sensors due to their attractive advantages over conventional electrical sensors, such as small sensing volume, good flexibility, real-time monitoring, remote operation, corrosion resistance, and immunity to EMI or RFI [5–9]. In particular, a number of fiber-optic temperature sensors and water level (or liquid level) detectors have been investigated using various optical sensing techniques, including light intensity modulation [10–15], wavelength shift [16–21], and optical image [22,23].

In this study, we used a special sensing element for simultaneous measurement of temperature and water level and developed a novel fiber-optic sensor system for SFP monitoring. Using an optical time-domain reflectometer (OTDR), the optical power of the reflected light due to the variation of the refractive index (*n*) was measured and analyzed to obtain temperature and water level information. OTDRs are commonly used to characterize the optical return loss and fiber length. However, OTDRs also can be used as a light-measuring device for employing distributed fiber-optic sensor [24–27]. By using an OTDR device and optical fibers, the proposed fiber-optic sensor transmits the temperature and water level information to a remote location where it is displayed.

**Figure 1.**
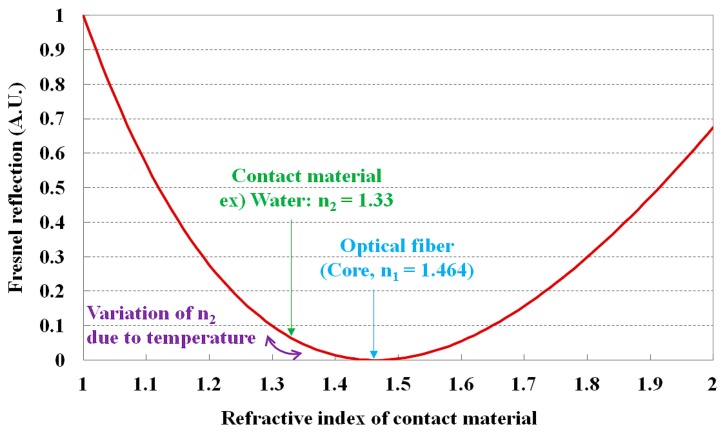
Relationship between the refractive index of the contact material and the Fresnel reflection when the core refractive index of optical fiber is 1.464.

To measure temperature and water level, we used Fresnel reflection, which causes a change in the reflection of light inside a sensing probe according to the temperature difference at the interface of water and air. The relationship between the refractive index of a contact material and the Fresnel reflection is shown in Figure 1. Generally, Fresnel reflection occurs at the coupling interface between two media with different refractive indices, and a portion of the light is reflected back into the first medium. Here, the fraction of optical power (*R*) that is reflected at the interface is given as [28]:

(1) $$R={\left(\frac{n_{1}-n_{2}}{n_{1}+n_{2}}\right)}^{2}$$

where *n*_1_ is the refractive index of the core of optical fiber and *n*_2_ is the refractive index of the contact material that makes contact with the core. By changing *n*_2_ with physical parameters, Fresnel reflection arising at the interface between the distal end of optical fiber and the contact material is also changed. With a decreasing difference between *n*_1_ and *n*_2_, the Fresnel reflection also decreases, as delineated in Equation (1) and Figure 1. Accordingly, the optical power of reflected light decreases while the transmission increases. If *n*_2_ can be varied by affecting certain physical parameters and it can modulate the optical properties of the light emitted from a light source, the contact material can be used as a sensing element to measure physical parameters.

## 2. Fabrication of a Sensing Probe

**Figure 2.**
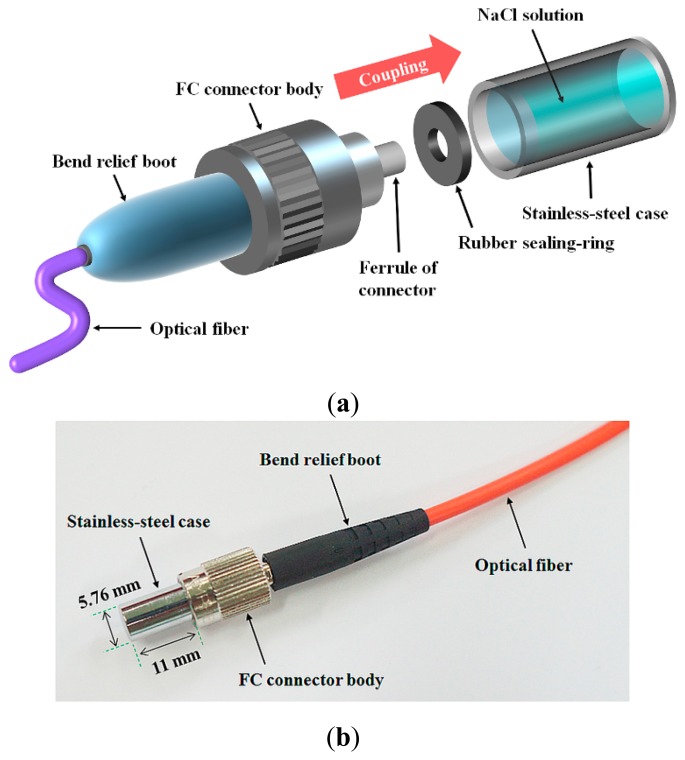
(**a**) Internal structure and (**b**) photograph of the sensing probe.

Figure 2 illustrates the structure of a sensing probe, which is composed of a sensing element, a stainless-steel case, a rubber sealing-ring, and an optical fiber. The temperature sensing type and water level sensing type probes have identical structure.

In our previous study, we have compared the sensitivity of two types of sensing probes using pure water and aqueous solution of sodium chloride (NaCl solution) according to the temperature change. As an experimental result, we have demonstrated that the thermal sensitivity of the NaCl solution-based sensing probe was higher than that of the sensing probe using pure water [29]. Therefore, we selected the transparent NaCl solution as a sensing element of the sensing probe in this study to measure temperature and water level simultaneously. The NaCl solution is inexpensive, affordable, and easy to handle. The NaCl solution was prepared by mixing NaCl powder (Sodium Chloride 99.5%, Wako Pure Chemical Industries, Ltd., Osaka, Japan) and distilled water. By changing temperature, the refractive index of the transparent NaCl solution is varied and then Fresnel reflection arising at the interface between the distal end of optical fiber and the NaCl solution is also changed; accordingly, it can modulate the optical property of the light transmitted from a light source.

In order to transmit a light signal modulated from a sensing probe, a single mode optical fiber (SMF: 980HP, Thorlabs, Inc., Newton, NJ, USA) was used. The diameter of the core is 3.6 μm and with the cladding the diameter is 125 ± 1.5 μm. The outside of the cladding is coated by ultraviolet (UV)-cured dual acrylate and thus the outer diameter of the fiber is 245 ± 15 μm. The operating temperature is from −55 to 85 °C. The refractive indices of the core and the cladding at the design wavelength of 980 nm are 1.4640 and 1.4507, respectively, and thereby the nominal numerical aperture (NA) is about 0.2. The SMF is transparent over a near-infrared (IR) spectral range ranging from 980 to 1600 nm. The short-term bend radius is longer than 6 mm and the attenuation is less than 3.5 dB/km at 980 nm. In this study, fiber-optic FC connectors were installed on both ends of SMF in order to easily and precisely connect with the sensing probe and a light-measuring device. Additionally, a mating sleeve was used to enable connection (and disconnection) of transmitting optical fiber with a length of 100 m to a sensing probe.

In fabricating the sensing probe, first, the NaCl solution was filled in a stainless-steel case with high thermal conductivity. Second, the gap at the coupling interface between the stainless-steel case and the ferrule of FC connector was sealed by a rubber sealing-ring. Finally, a fiber-optic FC connector was coupled with the stainless-steel case tightly, as depicted in Figure 2.

## 3. Fiber-Optic Sensor System

### 3.1. Fiber-Optic Temperature Sensor

**Figure 3.**
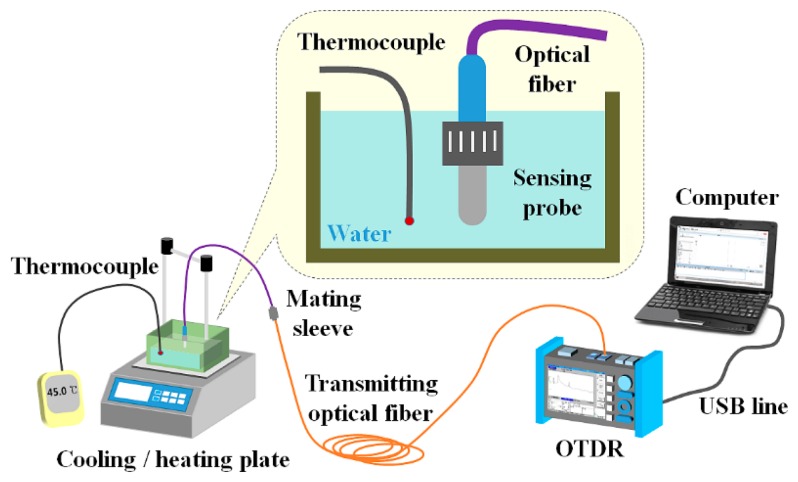
Composition of the fiber-optic temperature sensor to measure water temperature.

First, we fabricated a fiber-optic temperature sensor using a sensing probe as a basic sensor to develop a water level sensor. If only one probe is used in a fiber-optic sensor system, this system is specialized for remote measurement of contact temperature. Figure 3 shows the experimental setup employed for temperature measurement using the fiber-optic temperature sensor, which consists of a sensing probe, a transmitting optical fiber, a light-measuring device, and a laptop. As a light-measuring device with a built-in light source, we used a portable OTDR (AQ7275-735041, Yokogawa Electric Co., Tokyo, Japan), which can measure specific event signals and their locations at a long distance. An OTDR displays the distance *vs.* the optical power of the light signal returned by backscattering at the event location in the optical fiber. The OTDR used in this study has a short event dead-zone of 0.8 m, which enables detection of closely spaced events in optical fiber. In addition, it has a high dynamic range of up to 40 dB, which is effective in measuring long optical fibers and fiber-optic couplers (*i.e.*, splitters) with a large optical loss. The built-in light source has four different near-IR wavelengths, such as 850, 1300, 1310, and 1550 nm. Among these wavelengths, only 850 and 1300 nm are used for multimode optical fiber (MMF) and the other wavelengths can support SMF. Therefore, we employed a light source with wavelengths of 1310 and 1550 nm for measuring the optical power of the light returned via SMFs.

As can be seen in Figure 3, the sensing probe and a K-type thermocouple (54II thermometer, Fluke Co., Everett, WA, USA) were immersed in a water tank placed on a cooling/heating plate (CP-7200GT, Intec System, Seongnam, Korea). The temperature of the water was controlled by the temperature controller in the cooling/heating plate and monitored with a thermocouple. The light emitted from the light source in the OTDR was guided through the transmitting optical fiber to the sensing probe in the water, which was heated or cooled with the cooling/heating plate. The optical power of the light that reached the sensing probe was then changed by variation of Fresnel reflection in accordance with the temperature of the water. Finally, the modulated light reflected from the sensing probe was returned to the photodetector of the OTDR.

**Figure 4.**
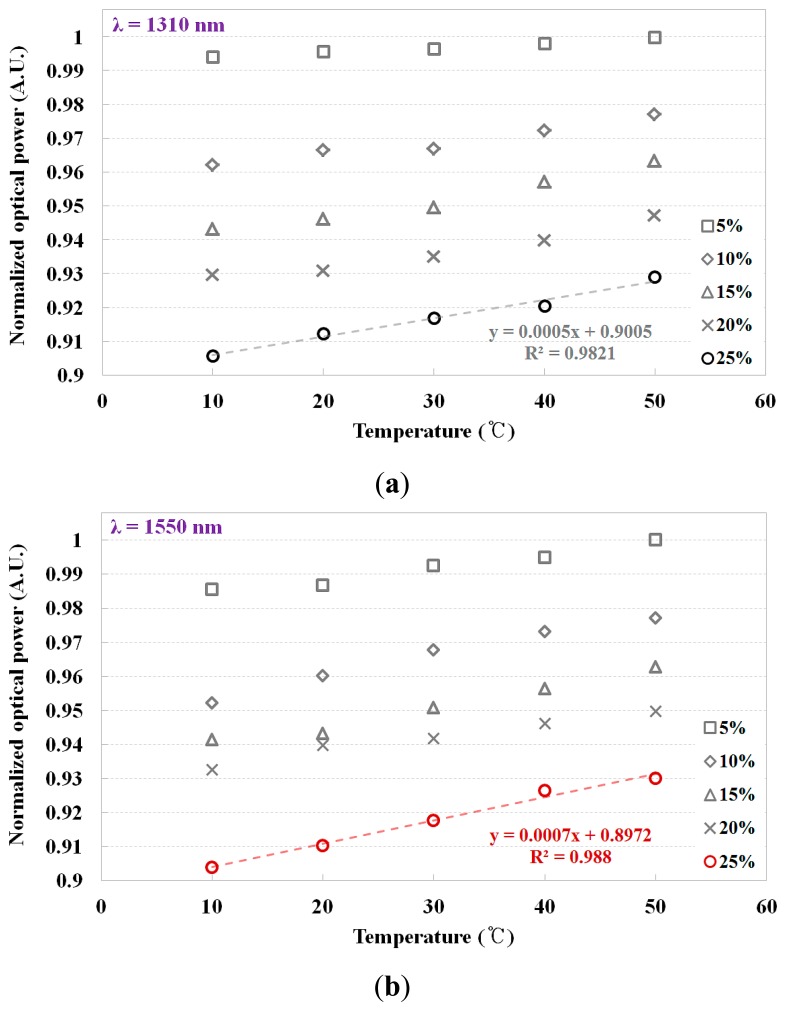
Comparison of the output signals of the fiber-optic temperature sensor with two wavelengths of the light source and five concentrations of the NaCl solution according to the temperature variation of the water. (**a**) λ = 1310 nm and (**b**) λ = 1550 nm.

Before the experimental study on regular thermometry using the fiber-optic temperature sensor, we selected the optimum peak wavelength (λ) of the light source and the optimum concentration (%) of the NaCl solution for optimization of the fiber-optic temperature sensor. Figure 4 shows the output signals (*i.e.*, optical power) of the fiber-optic temperature sensor according to the temperature variation of the water in the water tank when the concentration of the NaCl solution in the sensing probe was changed from 5% to 25%. It was reported that the refractive index of water decreased with the temperature rise and the refractive index of NaCl solution increased according to the concentration of NaCl [30,31]. By changing the temperature of the water or the concentration of NaCl, different refractive indices of the NaCl solution in the sensing probe are obtained. The response of the fiber-optic temperature sensor is dependent on the Fresnel reflection due to the change of the refractive index. As the temperature of the water was increased from 10 to 50 °C, the output signal of the fiber-optic temperature sensor also increased. When the concentration of the NaCl solution was 25%, we obtained a nearly linear relationship between the water temperature and the output signal of the fiber-optic sensor. Furthermore, the gradient of the curve became steeper when the peak wavelength of the light source was 1550 nm, as shown in Figure 4b. Therefore, using the experimental results of the feasibility study, we selected a NaCl solution with a concentration of 25% and a light source with a wavelength of 1550 nm.

**Figure 5.**
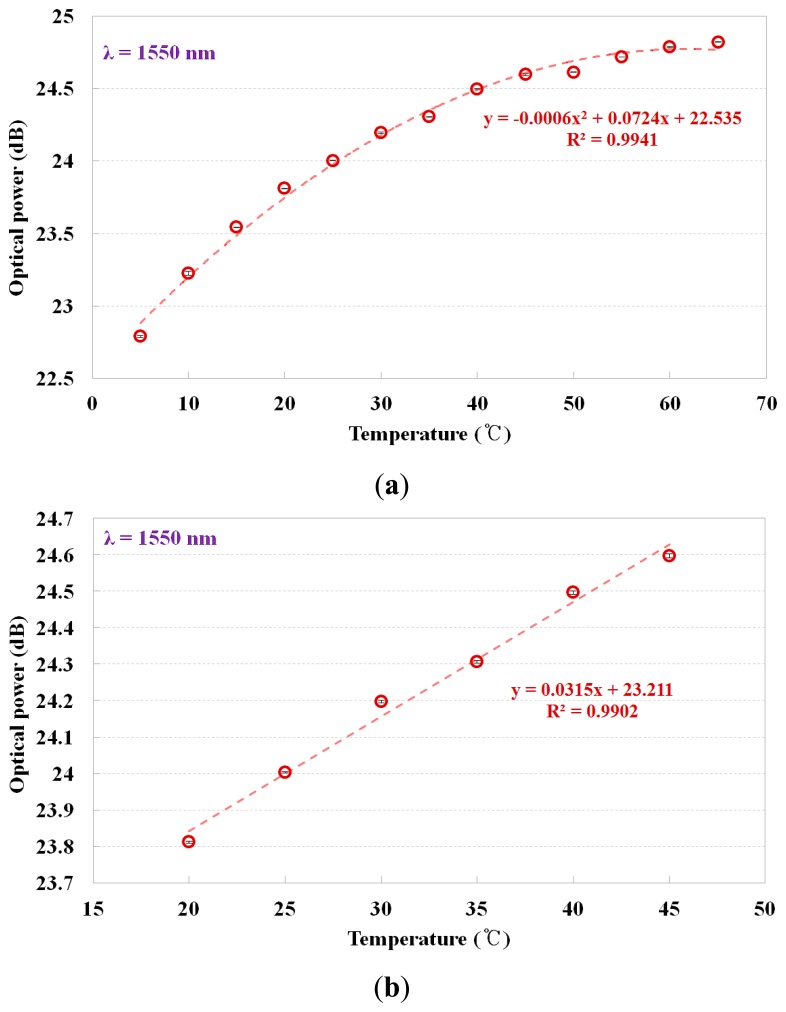
Relationship between the temperature of water and the optical power. (**a**) Water temperature in the broad range of 5 to 65 °C and (**b**) water temperature in the narrow range of 20 to 45 °C.

As can be seen in Figure 1, if the refractive index of a contact material becomes smaller than that of the core of the optical fiber, the Fresnel reflection at the interface between the optical fiber and the contact material increases and accordingly, the optical power of the reflected light also increased. Figure 5 describes the output signal of the fiber-optic temperature sensor *vs.* the temperature of the water, which was measured using a thermocouple, and the mathematical form of the best fit line to the curve is also presented. In this study, we measured the optical power (dB) through repeated and random experiments using the proposed sensor. Error bars were drawn on all data points in Figure 5; however, the error bars were generally within the data points because they were too small to be displayed. As the temperature of water was increased from 5 to 65 °C, the optical power of the reflected light gradually increased because the refractive index of the NaCl solution decreased.

In contrast to the results of Figure 4b, it can be seen that there is a non-linear dependence between the optical power and the water temperature in the relatively broad range of 5 to 65 °C, as shown in Figure 5a. However, it can be considered that the sensitivity to temperature is about 0.031 dB/°C in the linear response section because the fiber-optic temperature sensor has a nearly linear relationship between the optical power and the water temperature in the narrow range of 20 to 45 °C, as shown in Figure 5b. Consequently, the temperature of the water can be determined by the mathematical relation obtained by measuring the optical power of the light reflected from the sensing probes under the temperature condition of interest (under 60 °C) in the SFP during normal operation.

**Figure 6.**
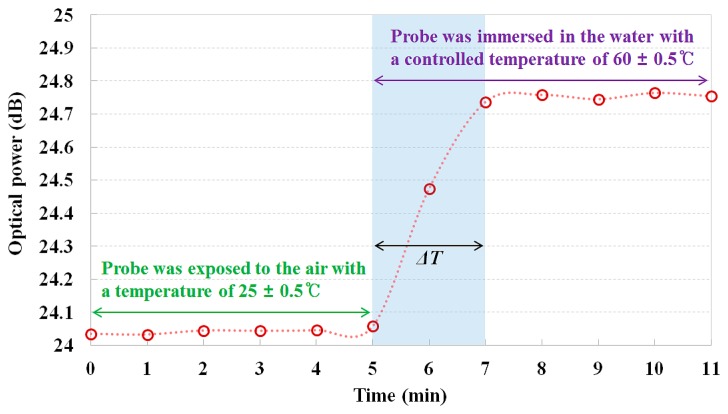
Real-time monitoring of the fiber-optic temperature sensor to measure response time.

The real-time monitoring results of the fiber-optic temperature sensor are shown in Figure 6. In this test, the sensing probe was exposed to the air with an ambient temperature of 25 °C and then quickly dipped into the water with a controlled temperature of 60 °C in the water tank. As expected, the optical power of the reflected light increased when the sensing probe was exposed to an environment of higher temperature. As a result, a response time (Δ*T*) of approximately 2 min was measured for a temperature variation of 35 °C between 25 °C and 60 °C. Therefore, the response time per degree Celsius could be calculated as about 3.43 s/°C. The response time of the proposed fiber-optic temperature sensor is substantially slower than that of the commercialized thermometer because the NaCl solution in the sensing probe has low thermal conductivity although it uses fast light signals. However, the proposed sensor is useful for monitoring temperature with immunity to EMI or RFI at very long distances although it has a slow response time.

### 3.2. Fiber-Optic Water Level Sensor

**Figure 7.**
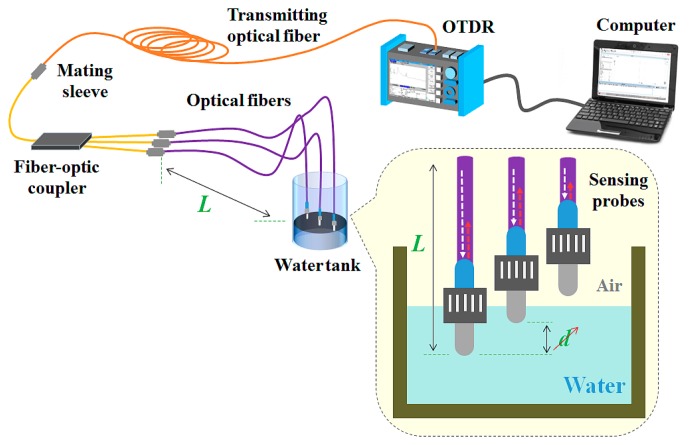
Experimental setup employed for the simultaneous measurement of temperature and water level using the fiber-optic water level sensor.

Next, we fabricated a prototype of the fiber-optic water level sensor with multiple sensing probes. Figure 7 shows the experimental setup using the fiber-optic water level sensor to measure temperature and water level simultaneously. In order to monitor the change of the water level inside a water tank, a number of identical sensing probes and fiber-optic couplers are required. In this study, a fiber-optic coupler (FCQ1315-FC, Thorlabs, Inc., Newton, NJ, USA) with 50 cm long input and output fibers was used to connect three sensing probes with different lengths (*L*) to a transmitting optical fiber. As noted in Section 2, the structure of the sensing probe to measure water level is identical to that of the temperature-sensing probe. In the case of the fiber-optic water level sensor with multiple sensing probes, its measurable distance is shorter than that of the fiber-optic temperature sensor because the water level sensor uses several optical fibers and couplers with a large optical loss. However, this sensor can measure water level, as well as temperature by incorporating additional sensing probes. Using the proposed fiber-optic water level sensor, the water level was determined by measuring the difference in temperature of individual sensing probes placed inside and outside of the water in a water tank.

**Figure 8.**
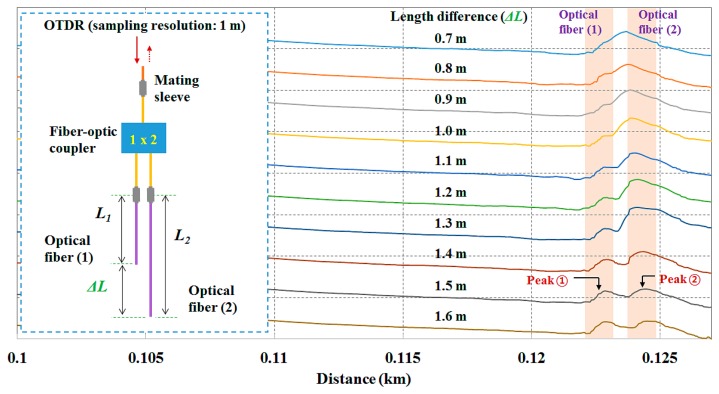
Variation of each optical peak with change in the length difference between the two optical fibers.

For simultaneous and multipoint measurements using the OTDR device, it is important to consider the length difference (*i.e.*, spatial interval) between the sensing probes although the OTDR used in this study has a short pulse width of 3 ns, which allows events that are close together to be measured separately. Figure 8 shows the variation of each optical peak according to the change of the length difference (Δ*L*) between the two optical fibers. In this test, two uncoupled optical fibers with the sensing probe were connected to the fiber-optic coupler and their distal ends were exposed to the air. We then measured the optical peaks of two light signals reflected at each end-surface of the two optical fibers having different lengths. When the length difference was shorter than 1.0 m, two optical peaks of each light signal overlapped. On the other hand, normal optical peaks having similar amplitude were observed when the length difference was greater than 1.5 m. In this study, we therefore fabricated three sensing probes using optical fibers whose length difference is more than 1.5 m.

**Figure 9.**
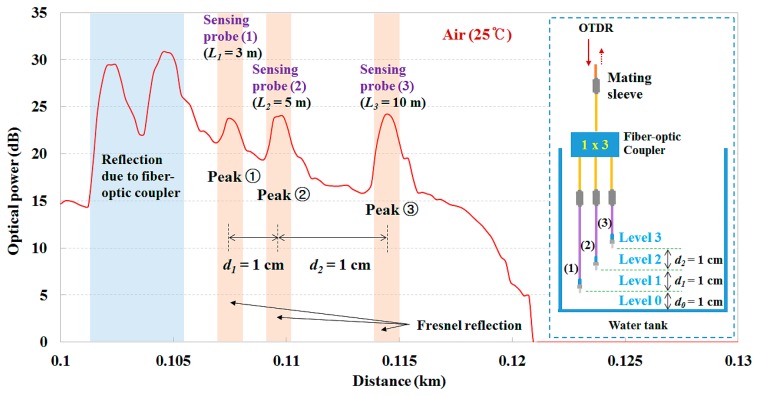
Individual light signals reflected from three sensing probes exposed to air with a temperature of 25 °C.

Figure 9 shows the light signals reflected from three sensing probes exposed to air with a temperature of 25 °C in the empty water tank. To obtain individual optical peaks, the lengths of each sensing probe were selected as 3, 5, and 10 m, respectively, and, thereby, the length difference was greater than 1.5 m. For determination of the water level inside the water tank, the sensing probe (1) was installed at a height of 1 cm from the bottom of the water tank, whereas the others were distributed vertically. Here, the distance (*d*) between the distal ends of each sensing probe (*i.e.*, measuring points) is 1 cm. In order to change the measurement resolution, the location of each sensing probe can be easily changed in accordance with the measuring conditions. In addition, it is possible to increase the number of sensing probes considering the optical loss in the proposed sensor system.

**Figure 10.**
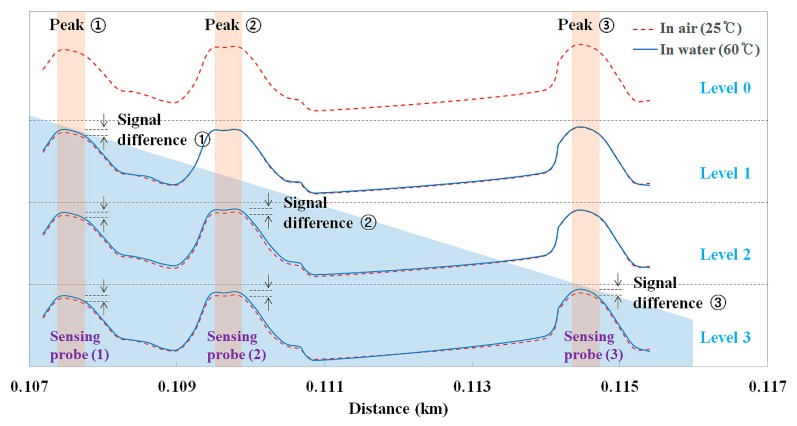
Variation of the output signals of the fiber-optic water level sensor according to the water level.

Figure 10 shows the variation of the output signals of the fiber-optic water level sensor according to the water level. The water tank is gradually filled until that last sensing probe is immersed in water (e.g., level 3, as shown in Figures 9 and 10) and the optical power of the light reflected from the sensing probe depends on whether water is present, as can be seen Figure 1. When the sensing probe is immersed in water with a temperature of 60 °C, the optical power is increased by the temperature rise. In other words, if the sensing probe makes contact with the water, the optical power of the optical peak shown as a red dotted line increases and becomes a blue solid line. Therefore, the water level can be determined by measuring the signal difference of individual sensing probes, which are placed inside and outside of the water, as shown in Figure 10. Furthermore, it was found that each light signal was measured individually and independently to obtain real-time water level information while the multiplexed array of sensing probes was connected to a fiber-optic coupler. On the basis of the experimental results, it was successfully demonstrated that the proposed fiber-optic sensor system can be used to measure temperature and water level at a long distance.

## 4. Conclusions

We developed a prototype of a fiber-optic sensor system using a multiplexed array of NaCl solution-based sensing probes for application in a SFP. The proposed sensor system has very simple structure, affordable components, easy measuring principle, immunity to EMI or RFI, and confident response to measure key environment variables of the SFP at very long distances. First, we fabricated a fiber-optic temperature sensor with a sensing probe and measured the optical power due to the Fresnel reflection as a function of temperature. Fresnel reflection could modulate the optical power of the light reflected in the sensing probe according to the temperature of the water. From the experimental results, the relationship between the temperature of water and the output signal of the fiber-optic temperature sensor was obtained. Using the mathematical relation, we can determine the temperature of the water by measuring the optical power of the light reflected from the sensing probes in a temperature range of 5 to 65 °C. Next, we fabricated a fiber-optic water level sensor using three sensing probes and a fiber-optic coupler. This sensor can provide water level information with temperature by incorporating additional sensing probes. In this study, the water level was simply determined by measuring the signal difference due to the temperature difference of two sensing probes placed inside and outside of the water, using the fabricated fiber-optic water level sensor. The precondition for operating this water level sensor is that the temperature of water must to be different from that of air. If the temperature of water reaches the same temperature as air, it causes ambiguity in the reading of the water level. Fortunately, however, the temperature of water in the SFP is almost always higher than the ambient temperature of the air because of the decay heat produced by the spent fuel.

In the proposed fiber-optic sensor system, a general-purpose OTDR device was used to measure the optical power of the light reflected from the sensing probe, and it has caused production cost of the sensor system to rise. However, an OTDR can be useful as a light-measuring device in order to apply various distributed fiber-optic sensors because it can measure both specific event signals and their locations at a very long distance by using a transmitting optical fiber and a fiber-optic coupler that can split or combine light signal with a low attenuation. Furthermore, the standardized components of the proposed sensor system, such as SMF, FC connector, mating sleeve, fiber-optic coupler, and portable OTDR, are commonly used in fiber-optic communications system, which is also installed in the various nuclear facilities including SFP and nuclear power plant, and thus almost components are affordable and easy to repair.

In conclusion, we demonstrate that temperature and water level can be obtained by measuring optical powers of light reflected from individual sensing probes based on a NaCl solution using a fiber-optic sensor system with an OTDR device. This approach makes it possible to remotely monitor the real-time change in the environment conditions, including temperature and water level, of the SFP. Based on the results of this study, it is expected that a fiber-optic sensor system can be used as an auxiliary monitoring system of the SFP during a loss of power accident, as well as in normal operation. Further studies will be carried out to fabricate a reliable fiber-optic sensor system with newly designed sensing probes to improve measurement sensitivity, response time, and measurable temperature range. A fiber-optic sensor will be designed and developed with an insulator and a shielding case to protect it from the high temperature and radiological environments under an extended loss of spent fuel cooling capacity.
